# RNAi targeting *Caenorhabditis elegans* α-arrestins has little effect on lifespan

**DOI:** 10.12688/f1000research.12337.4

**Published:** 2017-12-08

**Authors:** Sangsoon Park, Yoonji Jung, Seon Woo A. An, Heehwa G. Son, Wooseon Hwang, Dongyeop Lee, Murat Artan, Hae-Eun H. Park, Dae-Eun Jeong, Yujin Lee, Seung-Jae V. Lee

**Affiliations:** 1 Department of Life Sciences, Pohang University of Science and Technology, Pohang, Gyeongbuk, 37673, Korea, South; 2 School of Interdisciplinary Bioscience and Bioengineering, Pohang University of Science and Technology, Pohang, Gyeongbuk, 37673, Korea, South

**Keywords:** C. elegans, α-arrestin, insulin/IGF-1 signaling, aging, lifespan

## Abstract

**Background: **α-arrestins are a family of proteins that are implicated in multiple biological processes, including metabolism and receptor desensitization.

**Methods: **Here, we sought to examine the roles of α-arrestins in the longevity of *Caenorhabditis elegans* through an RNA interference screen.

**Results: **We found that feeding worms with bacteria expressing double-stranded RNA against each of 24 out of total 29 *C. elegans *α-arrestins had little effect on lifespan. Thus, individual *C. elegans* α-arrestins may have minor effects on longevity.

**Conclusions: **This study will provide useful information for future research on the functional role of α-arrestins in aging and longevity.

## Introduction

α-arrestins are a family of proteins that contain arrestin domains whose sequences and structures have similarities with those of classical visual and β-arrestins ^1– 3^. α-arrestins are considered as ancestral forms of arrestins because their orthologs exist in fungi, including yeast, which do not have visual or β-arrestins ^1, 4^. Several mammalian α-arrestins modulate metabolism and receptor desensitization ^5, 6^, but much remains to be elucidated concerning the functions of α-arrestins in many organisms. The human genome encodes 6 α-arrestins and 4 visual or β-arrestins. Interestingly, the simple roundworm *Caenorhabditis elegans* genome contains 29 α-arrestin and 1 β-arrestin genes ^1, 7^. Therefore, the *C. elegans* system provides opportunities for the genetic analysis of α-arrestins in various aspects of physiology both individually and combinatorially. However, information regarding the functions of *C. elegans* α-arrestins is limited ^8, 9^.

*C. elegans* is an excellent genetic model organism that has been exploited for studying conserved biological processes, including apoptosis, behavior, development and aging. In particular, its short lifespan in combination with genetic amenability has made *C. elegans* one of the most popular models for research on aging and longevity ^10, 11^. Many factors, including components in insulin/insulin-like growth factor 1 (IGF-1) signaling (IIS), have been identified as lifespan and aging regulators in *C. elegans* ^11– 13^. For example, genetic inhibition of IIS components, such as DAF-2/insulin/IGF-1 receptor, robustly extends lifespan and delays physiological aging through up-regulating transcription factors, including DAF-16/FOXO ^11– 13^. Importantly, the roles of these aging-regulatory factors in *C. elegans* have been shown to be conserved in other species, including *Drosophila* and mammals ^12, 13^. One of the powerful ways to identify novel factors that influence aging is by employing genetic screens, such as an RNA interference (RNAi) screen. We previously identified several genetic factors, including RNA helicases, that modulate longevity in *C. elegans*, through targeted or genome-wide RNAi screens ^14– 17^. Because of its robust longevity phenotype that confers sensitivity to changes in lifespan, *daf-2*/insulin/IGF-1 receptor mutants serve as an excellent platform for the identification of novel lifespan-regulating factors ^17, 18^.

In this study, we aimed to determine whether any α-arrestins played a role in the lifespan regulation of wild-type or *daf-2* mutants. We performed a lifespan assay-based RNAi screen targeting 24 out of 29 *C. elegans* α-arrestins. We found that α-arrestin double-stranded RNA (dsRNA)-expressing *E. coli* feeding had little effect on the lifespan of wild-type or *daf-2* mutants. Thus, *C. elegans* α-arrestins may play minor or modulatory roles in lifespan regulation. Based on our results, it will be important to test the roles of α-arrestins in combinatorial manners and/or by using strong loss-of-function mutations in future research.

## Methods

### *Caenorhabditis elegans* strains

All strains were maintained as previously described ^19^. The following strains were used in this study: N2 wild-type, CF1041 *daf-2(e1370) III* outcrossed six times to N2.

### Phylogenetic analysis

The protein sequences of 27 α-arrestins, except *arrd* (arrestin domain protein)- *20* and *arrd-21*, were obtained from Wormbase ( www.wormbase.org, version WS259). The protein sequences of *arrd-20* and *arrd-21* were obtained from Ensembl ( http://www.ensembl.org, release 89). The phylogenetic tree of 29 α-arrestins in *C. elegans* was generated using Clustal Omega ( http://www.ebi.ac.uk/Tools/msa/clustalo/) ^20^ and re-visualized using the Dendroscope 3 (version 3.5.9) ^21^. For the α-arrestins that have multiple isoforms, isoform a was used for the analysis.

### RNAi clones

Twenty one RNAi clones that target *C. elegans* α-arrestin genes were used from two commercial *C. elegans* RNAi feeding libraries. Specifically, RNAi clones targeting *arrd-2*, *arrd-6*, *arrd-7*, *arrd-8*, *arrd-9*, *arrd-10*, *arrd-13*, *arrd-16, arrd-18*, *arrd-23*, *arrd-24*, *arrd-25*, *arrd-28* and *ttm-2* (toxin-regulated targets of MAPK 2) were obtained from Ahringer laboratory library (Geneservice Ltd., Cambridge, UK), while those against *arrd-1*, *arrd-3*, *arrd-4*, *arrd-5*, *arrd-14*, *arrd-15* and *arrd-19* were from Vidal laboratory library (Source BioScience, Nottingham, UK). Three RNAi clones targeting *arrd-11*, *arrd-17*, and *arrd-26* were generated by molecular cloning using infusion recombinase (EZ-Fusion ^TM^ Cloning Kit, Enzynomics, Daejeon, South Korea). The N2 genomic DNA was obtained through the lysis of worms using proteinase K (Invitrogen, Carlsbad, CA, USA) and N2 complementary DNA was obtained from RNA extraction using RNAiso Plus (Takara, Shiga, Japan) followed by reverse transcription using ImProm-II reverse transcriptase (Promega, Madison, WI, USA, USA). The infusion reaction between PCR products and pL4440 plasmids (Fire lab *C. elegans* vector kit, 1999) digested with HindIII (New England Biolabs, Ipswich, MA, USA) and Acc65I (New England Biolabs) was followed by transformation of in-house competent *E. coli* HT115 cells and by selection of positive clones on ampicillin (USB, Santa Clara, CA, USA)-containing LB plates. Primers (CosmoGenetech, Seoul, South Korea) that were used to amplify coding regions of *arrd-11* from N2 genomic DNA, and those of *arrd-17* and *arrd-26* from N2 complementary DNA are as follows: forward primer 5’-GAATTCGATATCAAGCTCCCTCGTGCAAATTAGGAAA-3’ and reverse primer 5’-CTATAGGGCGAATTGGGGTTCCTCCCACTCCATACA-3’ for *arrd-11*; forward primer 5’-GAATTCGATATCAAGCTATGGTGCAGTTAGATCGTTTTG-3’ and reverse primer 5’-CTATAGGGCGAATTGGTTAATCGGTATAAAATGG-3’ for *arrd-17*; forward primer 5’-GAATTCGATATCAAGCTATGAAGGTCGATTACTTCG-3’ and reverse primer 5’- CTATAGGGCGAATTGGCTACTTCTCGGAGCCATTTG-3’ for *arrd*-26. The sequences of all these 24 α-arrestin RNAi clones were confirmed using DNA sequencing (Solgent, Daejeon, South Korea) before lifespan assays. RNAi clone for *arrd-12*, *arrd-22*, or *arrd-27* was not generated because we were not able to obtain transformed *E. coli* (HT115) colonies using *arrd-12* or *arrd-22* infusion reaction products, or the PCR product for *arrd-27* genomic or complementary DNA. *arrd-20* and *arrd-21* are predicted to be pseudogenes (Wormbase, version WS259) and were excluded from our screen. All RNAi clones used in our screen were examined for their potential off-target effects by using Clone Mapper ( http://bioinformatics.lif.univ-mrs.fr/RNAiMap) ^22^, and no significant off-target was predicted except *arrd-19* RNAi, which may additionally target a predicted pseudogene *arrd-21* ( Table S1). Experimental validation by qRT-PCR will be necessary to completely exclude the possible off-target effects caused by RNAi clones.

### RNAi screen using lifespan assay

The RNAi screen employing lifespan assay was performed as previously described ^17^. Briefly, *E. coli* HT115 bacteria that expressed specific dsRNAs were cultured overnight at 37°C in LB media containing 50 μg/ml ampicillin (USB). The cultured bacteria were seeded onto nematode growth media plates containing 50 μg/ml ampicillin and incubated overnight at 37°C. The seeded bacteria were treated with 1 mM isopropyl β-D-1-thiogalactopyranoside (Gold Biotechnology, St. Louis, MO, USA) and incubated at room temperature for approximately 24 h to induce dsRNAs. Age-synchronized wild-type N2 and *daf-2(e1370)* mutant animals were grown on RNAi plates from embryo to L4 stage at 20°C. Worms were then transferred onto RNAi plates containing 5 μM 5-fluoro-2’deoxyuridine (FUdR; Sigma, St. Louis, MO, USA), which prevents eggs from hatching, at young (day 1) adult stage, and transferred again to new FUdR-containing RNAi plates after 1 or 2 days. Eggs laid by *ttm-2* RNAi-treated worms hatched more frequently than control eggs on FUdR-containing plates in two independent lifespan experiments. All lifespan assays were performed at 20°C as duplicates by two independent researchers. The survival of worms was determined by gently touching worms with a platinum wire. Worms that did not respond were counted as dead worms and removed from the plates. Worms that crawled off the plates, burrowed into the agar media, or displayed internal hatching or vulval rupture were censored, but included in the statistical analysis. Lifespan data from two independent lifespan experiments within the experimental sets were pooled and used for statistical analysis (see Dataset 1– Dataset 3). OASIS 2 (Online Application for Survival Analysis 2; https://sbi.postech.ac.kr/oasis2) ^23^ was used for the statistical analysis of lifespan results. *P* values were calculated using long-rank (Mantel-Cox method) test.

## Results

### Feeding bacteria expressing dsRNA against each of several *C. elegans* α-arrestins marginally influenced lifespan

**Figure 1.**
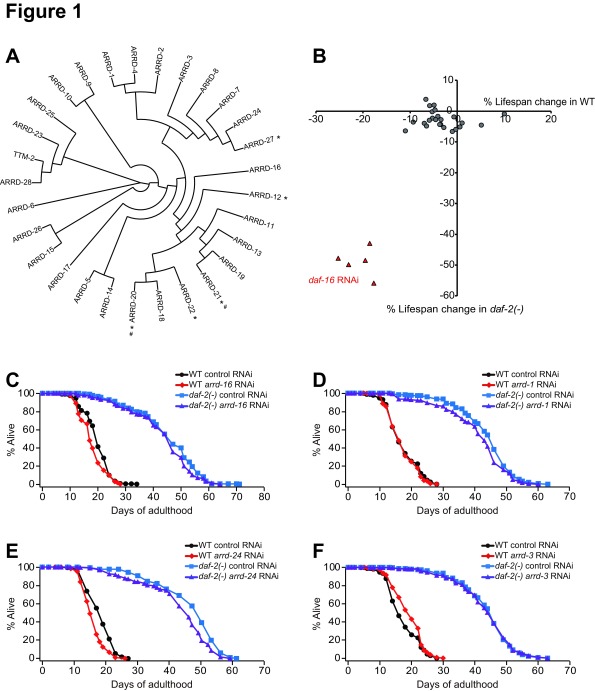
Feeding bacteria expressing dsRNA targeting each of *Caenorhabditis elegans* α-arrestins had little effect on lifespan. ( **A**) A phylogenetic tree showing *C. elegans* α-arrestin family members. Asterisks (*) indicate α-arrestins that were not examined in this study and number signs (#) indicate predicted pseudogenes. ( **B**) Each circle represents percent mean lifespan change by feeding wild-type (WT) and *daf-2(e1370)* ( *daf-2(-)*) mutants with bacteria expressing dsRNA against each of α-arrestin genes. Red triangles indicate lifespan changes by *daf-16* RNAi bacteria feeding, which was used as a positive control. ( **C**– **F**) Lifespan curves of WT and *daf-2(-)* animals fed with dsRNA-expressing bacteria targeting *arrd-16* ( **C**), *arrd-1* ( **D**), *arrd- 24* ( **E**), and *arrd-3* ( **F**). See Table S2 for statistical analysis.

We measured the lifespan of wild-type and long-lived *daf-2* mutant animals fed with bacteria expressing dsRNA targeting each of 24 of 29 genes encoding putative α-arrestin proteins ( Figure 1A). We used *daf-16* RNAi that largely suppressed the longevity of *daf-2(-)* mutants as a positive control ( Figure 1B, Figure S1A, Table S2) ^17, 18^. We found that feeding bacteria expressing dsRNA against specific α-arrestin genes caused a minor reduction of lifespan in wild-type or in *daf-2(-)* mutants ( Figure 1B, Figure S1B–U, Table S2). Out of the 24 RNAi clones, bacteria expressing dsRNA targeting *arrd* (arrestin domain protein) *-13*, *arrd-16*, *arrd-23*, *arrd-24* or *arrd-25* in wild-type, and bacteria expressing dsRNA against *arrd-1*, *arrd-2*, *arrd-5*, *arrd-24* or *arrd-28* in *daf-2(-)* mutant animals decreased lifespan by more than 5% ( Figure 1B–E, Figures S1B, D, K, Q, R, T, Table S2). Specifically, bacteria expressing dsRNA against *arrd-16* decreased lifespan in wild-type by 9%, and bacteria expressing dsRNA against *arrd-1* decreased lifespan in *daf-2(-)* mutants by 7% ( Figure 1B and C). In addition, bacteria expressing dsRNA targeting *arrd-24* decreased the lifespan of wild-type and *daf-2* mutant animals by 11% and 6%, respectively ( Figure 1E). In contrast, bacteria expressing dsRNA against *arrd-3* increased the lifespan of wild-type animals by 10% ( Figure 1F). Overall, feeding *C. elegans* with bacteria expressing dsRNA against individual α-arrestin genes appears to have minor effects on lifespan.

Kaplan-Meier estimator of RNAi lifespan experiments Kaplan-Meier estimate values were calculated from pooled lifespan data of two independent lifespan experiments using OASIS2 ( https://sbi.postech.ac.kr/oasis2/). ‘At risk’ indicates the number of individuals at risk just prior to a specific time point. ‘S_hat’ indicates Kaplan-Meier estimate of survival function, ‘Var S_hat’ indicates variance of ‘S_hat’, and ‘SE(S)’ indicates standard error of ‘S_hat’. Click here for additional data file. Copyright: © 2017 Park S et al. 2017 Data associated with the article are available under the terms of the Creative Commons Zero "No rights reserved" data waiver (CC0 1.0 Public domain dedication).

Mean lifespan and mortality rates of RNAi experiment results Mean lifespan, ages at different percent mortality, linear interpolation of mortality curve at specific mortality rate were calculated from pooled lifespan data of two independent lifespan experiments. Data were obtained using OASIS2 ( https://sbi.postech.ac.kr/oasis2/). Click here for additional data file. Copyright: © 2017 Park S et al. 2017 Data associated with the article are available under the terms of the Creative Commons Zero "No rights reserved" data waiver (CC0 1.0 Public domain dedication).

Statistical analysis of lifespan data Statistical test results (Chi square, *p* value, Bonferroni *p* value) were calculated between ‘condition 1’ and ‘condition 2’. Test results were obtained using OASIS2 ( https://sbi.postech.ac.kr/oasis2/). Click here for additional data file. Copyright: © 2017 Park S et al. 2017 Data associated with the article are available under the terms of the Creative Commons Zero "No rights reserved" data waiver (CC0 1.0 Public domain dedication).

## Discussion

The lifespan-regulatory functions of α-arrestins remain largely unexplored at the organism level. Here we showed that feeding bacteria expressing dsRNA targeting individual *C. elegans* α-arrestins had little effect on lifespan in wild-type or *daf-2* mutants. Our study has limitations that need to be considered for interpretation. First, feeding RNAi targeting some α-arrestins might be insufficient for causing strong lifespan phenotypes. This may be because many of *C. elegans* α-arrestins are predicted to be expressed in neurons ^7, 9, 24– 26^, which are refractory to RNAi ^27– 29^. Lifespan assays using RNAi-hypersensitive mutants, including *rrf-3(-)* and *eri-1(-)* animals ^29– 31^, treated with α-arrestin RNAi, or using α-arrestin null mutants will help address this issue. In addition, as we did not test whether RNAi targeting each α-arrestin gene was effective by using quantitative RT-PCR, our negative data should be interpreted with caution. Second, *C. elegans* α-arrestins may have functional redundancy, considering the large number of the α-arrestin family members in *C. elegans* and their sequence similarity ^1, 7^, which may obscure examining the functional importance of each α-arrestin. In addition, some α-arrestins may mostly function by modulating the action of their interacting proteins ^6^. In this case, genetic inhibition of α-arrestins may rather subtly affect the functions of their interacting partners that directly regulate physiology, such as aging and longevity, causing weak or no phenotypes. Thus, it will be interesting to test the effects of simultaneous inhibition of α-arrestins possibly through targeting the arrestin domain, and to identify and to functionally characterize proteins that bind *C. elegans* α-arrestins. Third, it is possible that three α-arrestins, *arrd-12*, *arrd-22* and *arrd-27*, which were not tested in our screen, may play crucial roles in lifespan regulation. Thus, it will be important to examine if genetic inhibition of each of these three α-arrestin genes affects lifespan in future studies.

In mammals, several α-arrestins are implicated in metabolic regulation ^5^. TXNIP (thioredoxin-interacting protein), an inhibitor of thioredoxin in mammals ^32– 34^, is a crucial negative regulator of glucose uptake ^35, 36^. ARRDC4 inhibits glucose uptake in cultured mammalian cells as well ^36^, and ARRDC3 deficiency protects against obesity in male mice through increasing energy expenditure ^37^. Because metabolism is closely associated with aging ^38^, it will be interesting to test whether α-arrestins in complex metazoans play roles in aging via regulating metabolism.

## Data availability

The data referenced by this article are under copyright with the following copyright statement: Copyright: © 2017 Park S et al.

Data associated with the article are available under the terms of the Creative Commons Zero "No rights reserved" data waiver (CC0 1.0 Public domain dedication). 

**Dataset 1. Kaplan-Meier estimator of RNAi lifespan experiments.** Kaplan-Meier estimate values were calculated from pooled lifespan data of two independent lifespan experiments using OASIS2 ( https://sbi.postech.ac.kr/oasis2/). ‘At risk’ indicates the number of individuals at risk just prior to a specific time point. ‘S_hat’ indicates Kaplan-Meier estimate of survival function, ‘Var S_hat’ indicates variance of ‘S_hat’, and ‘SE(S)’ indicates standard error of ‘S_hat’. doi, 10.5256/f1000research.12337.d173158 ^39^

**Dataset 2. Mean lifespan and mortality rates of RNAi experiment results.** Mean lifespan, ages at different percent mortality, linear interpolation of mortality curve at specific mortality rate were calculated from pooled lifespan data of two independent lifespan experiments. Data were obtained using OASIS2 ( https://sbi.postech.ac.kr/oasis2/). doi, 10.5256/f1000research.12337.d173159 ^40^

**Dataset 3. Statistical analysis of lifespan data.** Statistical test results (Chi square, *p* value, Bonferroni *p* value) were calculated between ‘condition 1’ and ‘condition 2’. Test results were obtained using OASIS2 ( https://sbi.postech.ac.kr/oasis2/). doi, 10.5256/f1000research.12337.d173160 ^41^

## Supplementary material

**Figure S1. Feeding bacteria expressing dsRNA against each *Caenorhabditis elegans* α-arrestin had minor effects on the lifespan of wild-type or *daf-2(-)* mutants.** ( **A**– **U**). Lifespan curves of wild-type (WT) and *daf-2(e1370)* ( *daf-2(-)*) mutants fed with bacteria expressing dsRNA targeting *daf-16* ( **A**), which was used as a positive control, *arrd-2* ( **B**), *arrd-4* ( **C**), *arrd-5* ( **D**), *arrd-6* ( **E**), *arrd-7* ( **F**), *arrd-8* ( **G**), *arrd-9* ( **H**), *arrd-10* ( **I**), *arrd-11* ( **J**), *arrd-13* ( **K**), *arrd-14* ( **L**), *arrd-15* ( **M**), *arrd-17* ( **N**), *arrd-18* ( **O**), *arrd-19* ( **P**), *arrd-23* ( **Q**), *arrd-25* ( **R**), *arrd-26* ( **S**), *arrd-28* ( **T**) and *ttm-2* ( **U**). See Table S2 for statistical analysis and for additional repeats of lifespan data using *daf-16* RNAi.

**Table S1. Possible RNAi target prediction.** RNAi clones used in the screen were examined for their possible off-targets by Clone Mapper ( http://bioinformatics.lif.univ-mrs.fr/RNAiMap) ^22^. For RNAi clone names, ‘mv’ indicates an RNAi clone from Vidal library and ‘sjj’ indicates an RNAi clone from Ahringer library. RNAi clones generated by us for this study are labeled as ‘Lee lab’. Scores for potential target transcripts were calculated as described previously ^22^ and were rounded off to the second digit after the decimal point.

**Table S2. Lifespan data of *Caenorhabditis elegans* fed with α-arrestin dsRNA-expressing bacteria.** Lifespan data within the solid lines indicate the pooled experimental datasets that are performed at the same time by two independent researchers. *P* values were calculated within the sets using log-rank (Mantel-Cox) method ^23^. Percent (%) changes and *p* values for the lifespan data of wild-type (WT) and *daf-2(e1370)* worms fed with specific α-arrestin dsRNA-expressing bacteria were calculated against those of WT and *daf-2(e1370)* worms fed with control dsRNA-expressing bacteria within the sets, respectively. Percent (%) changes and *p* values for the lifespan data of *daf-2(e1370)* mutants fed with control dsRNA-expressing bacteria were calculated against those of WT worms fed with control dsRNA-expressing bacteria. SEM represents standard error of the mean, and 75th percentile indicates the age (days) at 75% mortality.
