# Supplementary material for: RNAi targeting Caenorhabditis elegans α-arrestins has little effect on lifespan
Source: F1000Res. 2017 Dec 8;6:1515. Originally published 2017 Aug 18. [Version 4] doi: 10.12688/f1000research.12337.4 (PMC5657022; doi:10.12688/f1000research.12337.4)
Supplement: Supplementary file 5 [file f1000research-6-14574-s0004.tgz › 5aace534-5492-4d46-abc0-86def7e6ef34.pdf]

Supplementary Table S1. Possible RNAi target prediction

Candidate gene	RNAi clone name	Possible target transcript	Possible target gene	Score
arrd-1	mv _T04B8.e	T04B8.3	*arrd-1*	100
arrd-2	sjj_Y51B9A.4	Y51B9A.4	*arrd-2*	100
arrd-3	mv_M176.1	M176.1a	*arrd-3*	100
	mv_M176.1	M176.1b	*arrd-3*	100
arrd-4	mv_ZK938.2	ZK938.2	*arrd-4*	100
	mv_ZK938.2	ZK938.4	*arrd-5*	0.00
arrd-5	mv_ZK938.4	ZK938.4	*arrd-5*	100
	mv_ZK938.4	ZK938.2	*arrd-4*	0.00
arrd-6	sjj_F40F8.8	F40F8.8a	*arrd-6*	100
	sjj_F40F8.8	F40F8.8b	*arrd-6*	100
arrd-7	sjj_Y17G7B.11	Y17G7B.11	*arrd-7*	100
arrd-8	sjj_Y17G7B.14	Y17G7B.14	*arrd-8*	100
	sjj_Y17G7B.14	K09A9.1	*nipi-3*	0.00
arrd-9	sjj_F15A4.9	F15A4.9	*arrd-9*	100
arrd-10	sjj_F58G1.6	F58G1.6	*arrd-10*	100
	sjj_F58G1.6	Y34F4.5a		0.00
	sjj_F58G1.6	Y18H1A.10		0.00
	sjj_F58G1.6	C38C3.4a		0.00
	sjj_F58G1.6	E02H9.6		0.00
	sjj_F58G1.6	D1069.2.2	*cpn-2*	0.00
arrd-11	Lee lab_R06B9.1	R06B9.1	*arrd-11*	100
	Lee lab_R06B9.1	R06B9.3	*arrd-13*	0.02
arrd-13	sjj_R06B9.3	R06B9.3	*arrd-13*	100
	sjj_R06B9.3	F35F10.12.1	*arrd-19*	1.10
	sjj_R06B9.3	F35F10.12.2	*arrd-19*	1.10
	sjj_R06B9.3	R06B9.1	*arrd-11*	0.05
	sjj_R06B9.3	T20D4.6	*arrd-22*	0.02
	sjj_R06B9.3	F35F10.11	*arrd-18*	0.00
	sjj_R06B9.3	C04E12.12	*arrd-21*	0.00
	sjj_R06B9.3	W09D6.1a		0.00
	sjj_R06B9.3	W09D6.1b		0.00
	sjj_R06B9.3	W09D6.1c		0.00
arrd-14	mv_R06B9.4	R06B9.4	*arrd-14*	100
arrd-15	mv_ZK643.1	ZK643.1a	*arrd-15*	100
	mv_ZK643.1	ZK643.1b	*arrd-15*	100
	mv_ZK643.1	ZK643.1c	*arrd-15*	100
	mv_ZK643.1	ZK643.1d	*arrd-15*	100
arrd-16	sjj_Y49E10.24	Y49E10.24a	*arrd-16*	100
	sjj_Y49E10.24	Y49E10.24b	*arrd-16*	43.35
	sjj_Y49E10.24	K11H12.1		0.18

	sjj_Y49E10.24	K11H12.9a		0.18
	sjj_Y49E10.24	Y95B8A.10a	*pde-6*	0.01
	sjj_Y49E10.24	Y49E10.15	*snr-6*	0.00
	sjj_Y49E10.24	Y54G2A.28a		0.00
arrd-17	Lee lab_ T12D8.4	T12D8.4	*arrd-17*	100
arrd-18	sjj_F35F10.11	F35F10.11	*arrd-18*	100
	sjj_F35F10.11	T20D4.6	*arrd-22*	0.73
	sjj_F35F10.11	C04E12.11	*arrd-20*	0.05
	sjj_F35F10.11	R06B9.3	*arrd-13*	0.00
	sjj_F35F10.11	F35F10.12.1	*arrd-19*	0.00
	sjj_F35F10.11	F35F10.12.2	*arrd-19*	0.00
arrd-19	mv_F35F10.12	F35F10.12.1	*arrd-19*	100
	mv_F35F10.12	F35F10.12.2	*arrd-19*	100
	mv_F35F10.12	C04E12.12	*arrd-21*	100
	mv_F35F10.12	R06B9.3	*arrd-13*	0.79
	mv_F35F10.12	T20D4.6	*arrd-22*	0.11
	mv_F35F10.12	C04E12.11	*arrd-20*	0.01
	mv_F35F10.12	R06B9.1	*arrd-11*	0.00
	mv_F35F10.12	F35F10.11	*arrd-18*	0.00
	mv_F35F10.12	W09D6.1a		0.00
	mv_F35F10.12	W09D6.1b		0.00
	mv_F35F10.12	W09D6.1c		0.00
arrd-23	sjj_T07F12.3	T07F12.3	*arrd-23*	100
arrd-24	sjj_F48F7.7	F48F7.7	*arrd-24*	100
	sjj_F48F7.7	F45E4.3a.1		0.00
	sjj_F48F7.7	F45E4.3a.2		0.00
arrd-25	sjj_C04C11.2	C04C11.2.1	*arrd-25*	100
	sjj_C04C11.2	C04C11.2.2	*arrd-25*	100
arrd-26	Lee lab_ R05C11.1	R05C11.1	*arrd-26*	100
arrd-28	sjj_F21A10.4	F21A10.4	*arrd-28*	100
ttm-2	sjj_F26G1.4	F26G1.4.1	*ttm-2*	100
	sjj_F26G1.4	F26G1.4.2	*ttm-2*	100
