# Supplementary material for: RNAi targeting Caenorhabditis elegans α-arrestins has little effect on lifespan
Source: F1000Res. 2017 Dec 8;6:1515. Originally published 2017 Aug 18. [Version 4] doi: 10.12688/f1000research.12337.4 (PMC5657022; doi:10.12688/f1000research.12337.4)
Supplement: Supplementary file 6 [file f1000research-6-14574-s0005.tgz › 1f2134f8-6d78-481a-b9f5-bc926821298d.pdf]

Supplementary Table S2. Lifespan data of *Caenorhabditis elegans* α-arrestin RNAi clones

Strain/treatment	Mean lifespan ±SEM (days)	75th percentile	% change	Number of animals that died/total	*p* value vs. control	Figure in text
WT/control RNAi	17.4±0.4	22		172/240		Fig. 1D, 1F, S1A, S1B, S1C, S1D
WT/*daf-16* RNAi	14.0±0.3	16	-20%	158/240	<0.0001	Fig. S1A
WT/*arrd-1* RNAi	17.3±0.3	20	-1%	158/240	0.4068	Fig. 1D
WT/*arrd-2* RNAi	17.5±0.3	20	+1%	171/240	0.7799	Fig. S1B
WT/*arrd-3* RNAi	19.2±0.3	23	+10%	182/240	0.0045	Fig. 1F
WT/*arrd-4* RNAi	18.3±0.4	23	+5%	168/240	0.0798	Fig. S1C
WT/*arrd-5* RNAi	17.4±0.3	20	0%	163/240	0.4941	Fig. S1D
daf-2(e1370)/control RNAi	43.7±0.7	49	+151% (vs. WT/ctrl RNAi)	147/210	<0.0001	Fig. 1D, 1F, S1A, S1B, S1C, S1D
daf-2(e1370)/*daf-16* RNAi	22.5±0.4	28	-48%	211/240	<0.0001	Fig. S1A
daf-2(e1370)/*arrd-1* RNAi	40.8±0.9	46	-7%	124/180	0.0274	Fig. 1D
daf-2(e1370)/*arrd-2* RNAi	41.5±0.7	46	-5%	175/210	0.0115	Fig. S1B
daf-2(e1370)/*arrd-3* RNAi	43.3±0.7	49	-1%	192/240	0.9633	Fig. 1F
daf-2(e1370)/*arrd-4* RNAi	41.7±0.7	49	-4%	203/240	0.1107	Fig. S1C
daf-2(e1370)/*arrd-5* RNAi	41.1±0.6	46	-6%	194/240	0.0026	Fig. S1D
WT/control RNAi	21.7±0.4	24		181/240		Fig. S1E, S1F, S1G, S1H, S1I
WT/*daf-16* RNAi	17.6±0.3	22	-19%	193/240	<0.0001	
WT/*arrd-6* RNAi	20.5±0.4	24	-5%	176/240	0.2356	Fig. S1E
WT/*arrd-7* RNAi	20.9±0.4	24	-3%	188/240	0.3993	Fig. S1F
WT/*arrd-8* RNAi	20.5±0.4	24	-5%	182/240	0.3324	Fig. S1G
WT/*arrd-9* RNAi	20.8±0.4	24	-4%	178/240	0.2675	Fig. S1H
WT/*arrd-10* RNAi	20.2±0.4	24	-7%	184/240	0.072	Fig. S1I
daf-2(e1370)/control RNAi	44.3±0.7	51	+105% (vs. WT/ctrl RNAi)	195/240	<0.0001	Fig. S1E, S1F, S1G, S1H, S1I
daf-2(e1370)/*daf-16* RNAi	25.3±0.4	30	-43%	197/241	<0.0001	

daf-2(e1370)/*arrd-6* RNAi	44.3±0.7	51	0%	194/240	0.4514	Fig. S1E
daf-2(e1370)/*arrd-7* RNAi	43.1±0.7	51	-3%	209/240	0.443	Fig. S1F
daf-2(e1370)/*arrd-8* RNAi	43.3±0.7	48	-2%	199/240	0.0516	Fig. S1G
daf-2(e1370)/*arrd-9* RNAi	43.6±0.7	51	-2%	207/240	0.0729	Fig. S1H
daf-2(e1370)/arrd-10 RNAi	46.0±0.8	55	+4%	207/240	0.0158	Fig. S1I
WT/control RNAi	19.8±0.3	24		201/240		Fig. 1C, S1K, S1L, S1M, S1O
WT/*daf-16* RNAi	14.8±0.2	17	-25%	205/240	<0.0001	
WT/*arrd-13* RNAi	18.7±0.3	22	-6%	186/210	0.003	Fig. S1K
WT/*arrd-14* RNAi	19.1±0.3	22	-4%	193/240	0.0509	Fig. S1L
WT/*arrd-15* RNAi	18.9±0.3	22	-5%	214/240	0.0831	Fig. S1M
WT/*arrd-16* RNAi	18.0±0.3	20	-9%	209/240	0.0001	Fig. 1C
WT/*arrd-18* RNAi	18.9±0.3	22	-5%	214/240	0.1381	Fig. S1O
daf-2(e1370)/control RNAi	44.9±0.9	53	+126% (vs. WT/ctrl RNAi)	172/210	<0.0001	Fig. 1C, S1K, S1L, S1M, S1O
daf-2(e1370)/*daf-16* RNAi	23.5±0.3	26	-48%	202/240	<0.0001	
daf-2(e1370)/arrd-13 RNAi	45.7±0.9	53	+2%	152/210	0.4607	Fig. S1K
daf-2(e1370)/arrd-14 RNAi	42.9±0.8	50	-4%	165/240	0.0433	Fig. S1L
daf-2(e1370)/arrd-15 RNAi	45.8±0.7	51	+2%	187/240	0.8875	Fig. S1M
daf-2(e1370)/arrd-16 RNAi	43.3±0.8	51	-4%	187/240	0.1174	Fig. 1C
daf-2(e1370)/arrd-18 RNAi	45.1±1.0	53	0%	145/180	0.486	Fig. S1O
WT/control RNAi	18.0±0.3	21		220/240		Fig. 1E, S1Q, S1R, S1S, S1T
WT/*daf-16* RNAi	14.9±0.2	17	-18%	206/240	<0.0001	
WT/*arrd-23* RNAi	16.8±0.3	19	-7%	210/240	0.008	Fig. S1Q
WT/*arrd-24* RNAi	16.1±0.2	18	-11%	212/240	<0.0001	Fig. 1E
WT/*arrd-25* RNAi	16.9±0.3	19	-7%	202/240	0.0022	Fig. S1R
WT/*arrd-26* RNAi	17.2±0.3	19	-5%	221/240	0.1342	Fig. S1S
WT/*arrd-28* RNAi	17.5±0.3	21	-3%	221/240	0.2188	Fig. S1T
daf-2(e1370)/control RNAi	45.9±0.8	53	+155% (vs. WT/ctrl RNAi)	201/210	<0.0001	Fig. 1E, S1Q, S1R, S1S, S1T
daf-2(e1370)/*daf-16* RNAi	20.3±0.4	24	-56%	206/240	<0.0001	
daf-2(e1370)/arrd-23 RNAi	44.2±0.7	52	-4%	228/240	0.0039	Fig. S1Q
daf-2(e1370)/arrd-24 RNAi	43.0±0.8	50	-6%	193/211	<0.0001	Fig. 1E
daf-2(e1370)/arrd-25 RNAi	43.7±0.6	50	-5%	211/240	<0.0001	Fig. S1R
daf-2(e1370)/arrd-26 RNAi	44.7±0.6	52	-3%	223/241	0.0006	Fig. S1S
daf-2(e1370)/arrd-28 RNAi	43.5±0.9	50	-5%	137/150	<0.0001	Fig. S1T

WT/control RNAi	20.3±0.4	24		184/240		Fig. S1J, S1N, S1P, S1U
WT/*daf-16* RNAi	15.6±0.4	18	-23%	175/240	<0.0001	
WT/*arrd-11* RNAi	20.2±0.4	25	-1%	194/240	0.7707	Fig. S1J
WT/*arrd-17* RNAi	20.5±0.4	24	+1%	187/240	0.9914	Fig. S1N
WT/*arrd-19* RNAi	19.6±0.4	24	-4%	183/240	0.1891	Fig. S1P
WT/*ttm-2* RNAi	19.1±0.4	24	-6%	171/240	0.0729	Fig. S1U
daf-2(e1370)/control RNAi	43.0±0.7	50	+112% (vs. WT/ctrl RNAi)	190/240	<0.0001	Fig. S1J, S1N, S1P, S1U
daf-2(e1370)/*daf-16* RNAi	21.5±0.4	25	-50%	199/243	<0.0001	
daf-2(e1370)/arrd-11 RNAi	42.2±0.7	50	-2%	175/210	0.3156	Fig. S1J
daf-2(e1370)/arrd-17 RNAi	41.3±0.6	46	-4%	199/240	0.0156	Fig. S1N
daf-2(e1370)/arrd-19 RNAi	41.1±0.6	46	-4%	201/240	0.0007	Fig. S1P
daf-2(e1370)/ttm-2 RNAi	41.7±0.6	49	-3%	200/240	0.0041	Fig. S1U
