# Supplementary material for: RNAi targeting Caenorhabditis elegans α-arrestins has little effect on lifespan
Source: F1000Res. 2017 Dec 8;6:1515. Originally published 2017 Aug 18. [Version 4] doi: 10.12688/f1000research.12337.4 (PMC5657022; doi:10.12688/f1000research.12337.4)
Supplement: Peer review history

# Peer review: Referee response for version 3

**DOI:** 10.5256/f1000research.14215.r27946

**Published:** 2017-11-15

**Authors:** Alessandro Bitto

**Affiliations:** 1 Department of Pathology, University of Washington, Seattle, Seattle, WA, USA

I appreciate the wish for the authors to move past this set of experiments and communicate their results to the field. Nevertheless, I must reiterate that, without testing the efficacy of their RNAi, their results cannot be interpreted, rather than be "interpreted with caution" as the authors suggest in the discussion.

 Indeed, the authors themselves agree in their response to my previous review that "providing evidence for RNAi targeting *arrd* genes is a key for concluding that knockdown of *arrd* genes does not affect lifespan."

 The authors suggest that performing qPCR on a new set of RNAi experiments would not demonstrate whether the knockdown worked on the worms followed for lifespan curves. While technically correct, I think it is fair to assume that their RNAi results are reproducible enough to warrant the assumption that whatever happens in a new set of RNAi experiments has happened in a previous one.

 The authors bring forth a third reason for not testing the efficiency of their RNAi by claiming that their results are still useful to the field as they are. In fact, as I have suggested in  my previous review, without qPCR testing of the efficacy of their RNAi, their results cannot be distinguished from the natural variation in lifespan of a control population of N2 worms, and are thus potentially meaningless.

 In all honesty, I do not think that performing 24 qPCR reactions is an unreasonable request, especially when it is agreed that it would be "key for concluding that knockdown of *arrd* genes does not affect lifespan", which is the main point of the manuscript.

I have read this submission. I believe that I have an appropriate level of expertise to confirm that it is of an acceptable scientific standard, however I have significant reservations, as outlined above.

# Response

**Published:** 2017-12-06

**Authors:** Seung-Jae V Lee

**Affiliations:** Department of Life Sciences, Pohang University of Science and Technology, Korea, South

Dear Dr. Alessandro Bitto,

We greatly appreciate your time and efforts for further valuable comments. We totally agree with your concern that we do not know the degree of RNAi knockdown efficiency in worms except *daf-16* RNAi, which was used as a positive control and showed expected lifespan phenotypes. Therefore, to precisely describe what we did, we changed “RNAi knockdown” to “feeding bacteria expressing dsRNA” throughout the manuscript. We sincerely thank you for fixing our misleading interpretation.

# Peer review: Referee response for version 2

**DOI:** 10.5256/f1000research.13946.r27143

**Published:** 2017-10-26

**Authors:** Alessandro Bitto

**Affiliations:** 1 Department of Pathology, University of Washington, Seattle, Seattle, WA, USA

The authors took effort to address most of the concerns we raised in our initial review, but did not address at all our major concern, apart from a note in the discussion about the limitations of their results.

 Thus our major concern stands: the authors must provide evidence that RNAi knockdown is functioning for each of the genes tested before their lifespan results can be interpreted correctly. Until such evidence is provided, there is no way of determining whether knockdown of individual arrestins really has limited effect on lifespan.

I have read this submission. I believe that I have an appropriate level of expertise to confirm that it is of an acceptable scientific standard, however I have significant reservations, as outlined above.

# Response

**Published:** 2017-11-02

**Authors:** Seung-Jae V Lee

**Affiliations:** Department of Life Sciences, Pohang University of Science and Technology, Korea, South

Dear Dr. Alessandro Bitto,

We greatly appreciate your comments and respect your concerns on our paper. We totally agree with your point that providing evidence for RNAi targeting *arrd* genes is a key for concluding that knockdown of *arrd* genes does not affect lifespan. Nevertheless, we think it is more beneficial for the research field and for our lab to publish our data as they are, instead of not publishing a paper or performing all the qRT-PCR experiments retrospectively because of the following reasons.

First, we emphasized the limitation and the pitfall of our data thoroughly and therefore the readers will be fully aware of that.

Second, even if we perform the *arrd* RNAi experiments again and measure mRNA levels now, we will not be able to know whether the RNAi worked for the ones that we used for our lifespan screen. In addition, we do not have a plan on working on *arrd* genes any more.

Third, with all these issues and limitations in mind, we think our negative data are still useful for the field. If some researchers want to do lifespan experiments for *arrd* genes in the future, they would rather use RNAi-sensitive strains and/or mutants by knowing our negative results. We think publishing these results is better for the field than keeping the data in our lab unpublished.

In any case, we sincerely appreciate your efforts for evaluating our manuscript.

# Peer review: Referee response for version 2

**DOI:** 10.5256/f1000research.13946.r25721

**Published:** 2017-10-24

**Authors:** Meng-Qiu Dong

**Affiliations:** 1 National Institute of Biological Sciences (NCBS), Beijing, China

In this paper, Park et al. knocked down 24 out of 29 alpha-arrestin genes in C. elegans and found that none of them produced a sizable effect on the lifespans of the wild type or the long-lived daf-2 worms. The methods used in this study were sound, and the data quality were up to standard with hundreds of worms assayed for RNAi of each alpha-arrestin gene. I think the work is honest, and the negative results in this paper are reliable.  

 Concerns:

 (1) I agree with the previous 2 reviewers that it is strange to leave out 5 members of the C. elegans  alpha-arrestin genes. 

 (2) I think it is fair to say that none of the 24 genes extended lifespan when knocked down. The statistically significant 10% increase of wild-type lifespan by arrd-3 RNAi is negligible,  given the variation of the lifespan assay. The relatively small p-value (0.0045) is blessed by the large sample size (n = 240). I wonder what the p-values would be if the two independent lifespan experiments had not been pooled. 

 (3) I would like the authors to report how many worms were censored.  

 (4) I wonder whether the authors had observed any noticeable phenotypes from their RNAi experiments, in particular, egg-laying defects. Strong egg-laying defects are easy to detect. G-protein signaling and beta-arrestin regulate the egg-laying behavior.

I have read this submission. I believe that I have an appropriate level of expertise to confirm that it is of an acceptable scientific standard.

# Response

**Published:** 2017-11-02

**Authors:** Seung-Jae V Lee

**Affiliations:** Department of Life Sciences, Pohang University of Science and Technology, Korea, South

Dear Dr. Meng-Qiu Dong,

We sincerely thank you for your valuable comments on our manuscript. We admit the limitations of our research and revised our manuscript to downplay our results so as not to confuse the readers. We addressed your concerns and revised our manuscript as described below.

In this paper, Park et al. knocked down 24 out of 29 alpha-arrestin genes in *C. elegans* and found that none of them produced a sizable effect on the lifespans of the wild type or the long-lived *daf-2* worms. The methods used in this study were sound, and the data quality were up to standard with hundreds of worms assayed for RNAi of each alpha-arrestin gene. I think the work is honest, and the negative results in this paper are reliable. 

Concerns:

(1) I agree with the previous 2 reviewers that it is strange to leave out 5 members of the *C. elegans* alpha-arrestin genes.

> We appreciate your comments. We leave out *arrd-20* and *arrd-21* because they are predicted to be pseudogenes (Wormbase, version WS259) that are expected not to encode functional proteins. In addition, we did not test *arrd-12*, *arrd-22* or *arrd-27* because we were not able to obtain the corresponding RNAi clones during our cloning. We addressed this issue in Methods and discussed potential lifespan-regulatory roles of those genes in Discussion as follows,

In Methods, RNAi clones: “RNAi clone for *arrd-12*, *arrd-22*, or *arrd-27* was not generated because we were not able to obtain transformed *E. coli* (HT115) colonies using *arrd-12* or *arrd-22* infusion reaction products, or the PCR product for *arrd-27* genomic or complementary DNA.”

In Discussion: “Third, it is possible that three α-arrestins, *arrd-12*, *arrd-22* and *arrd-27*, which were not tested in our screen, may play crucial roles in lifespan regulation. Thus, it will be important to examine if genetic inhibition of each of these three α-arrestin genes affects lifespan in future studies.”

(2) I think it is fair to say that none of the 24 genes extended lifespan when knocked down. The statistically significant 10% increase of wild-type lifespan by *arrd-3* RNAi is negligible, given the variation of the lifespan assay. The relatively small p-value (0.0045) is blessed by the large sample size (n = 240). I wonder what the p-values would be if the two independent lifespan experiments had not been pooled.

> We appreciate your valuable comments. We agree with you that the extent of lifespan changes by several *arrd* RNAi clones was small but statistically significant because of the large sample size. Even if the lifespan data are not pooled, many independent lifespan results for *arrd* RNAi clones were again statistically significant. In either case, pooled or not pooled, we think the main point of our data indicates little effects of *arrd* RNAi on lifespan.

(3) I would like the authors to report how many worms were censored. 

> We appreciate your comments. We provided raw lifespan data that contain the number of censored worms on each day of counting in Dataset1. One can also easily know the total number of censored worms by the difference between the number of animals that died and the total number of worms in Supplementary Table S2.

(4) I wonder whether the authors had observed any noticeable phenotypes from their RNAi experiments, in particular, egg-laying defects. Strong egg-laying defects are easy to detect. G-protein signaling and beta-arrestin regulate the egg-laying behavior.

> We appreciate your comments. Because we focused on the lifespan phenotype of *arrd* RNAi, unfortunately we did not pay attention to other physiological phenotypes and might have missed egg-laying phenotypes caused by some *arrd* RNAi clones. It will be an important point for future research.

# Peer review: Referee response for version 2

**DOI:** 10.5256/f1000research.13946.r27142

**Published:** 2017-10-20

**Authors:** Roger Pocock

**Affiliations:** 1 Development and Stem Cells Program, Monash Biomedicine Discovery Institute, Department of Anatomy and Developmental Biology, Monash University, Melbourne, Vic, Australia

The authors have partially addressed my concerns. Below I suggest further changes.

1. Change title from 'RNAi targeting Caenorhabditis elegans α-arrestins has small or no effects on lifespan' to 'RNAi targeting Caenorhabditis elegans α-arrestins has little effect on lifespan'
2. Also change from 'small or no effects' to 'little effect' throughout the text.
3. The authors state that they did not study arrd-12, 22 and 27 because of 'unknown technical problems'. It would be helpful for the field to be more specific here. Could they not be amplified?
4. In the results paragraph the authors state that 'knockdown of each of individual α-arrestin genes tended to slightly reduce lifespan in wild-type or in daf-2(-) mutants'
  This is a little misleading. Change to 'knockdown of specific α-arrestin genes caused a minor reduction of lifespan in wild-type or in daf-2(-) mutants'
5. Change from 'In addition, as we did not test whether RNAi targeting each of α-arrestin genes was effective by using quantitative RT-PCR' to 'In addition, as we did not test whether RNAi targeting of each α-arrestin gene was effective by using quantitative RT-PCR'

I have read this submission. I believe that I have an appropriate level of expertise to confirm that it is of an acceptable scientific standard.

# Response

**Published:** 2017-11-02

**Authors:** Seung-Jae V Lee

**Affiliations:** Department of Life Sciences, Pohang University of Science and Technology, Korea, South

Dear Dr. Roger Pocock,

We greatly appreciate your comments on our manuscript. We further addressed your concerns and revised our manuscript as described below.

The authors have partially addressed my concerns. Below I suggest further changes.

1. Change title from 'RNAi targeting *Caenorhabditis elegans* α-arrestins has small or no effects on lifespan' to 'RNAi targeting *Caenorhabditis elegans* α-arrestins has little effect on lifespan'

> We appreciate your comment on the title. We changed the title as follows,

Title: RNAi targeting *Caenorhabditis elegans *α-arrestins has little effect on lifespan

2. Also change from 'small or no effects' to 'little effect' throughout the text.

> We also changed the ‘small or no effects’ to ‘little effect’ in the main text as follows,

In Abstract: “We found that knocking down each of 24 out of total 29 *C. elegans* α-arrestins had little effect on lifespan.”

In Introduction: “We found that knocking down each of the α-arrestins had little effect on the lifespan of wild-type and *daf-2* mutants.”

In Discussion: “Here we showed that RNAi knockdown of individual *C. elegans* α-arrestins had little effect on lifespan in wild-type or *daf-2* mutants.”

In Figure legend: “Figure 1. RNAi targeting each of *Caenorhabditis elegans* α-arrestins had little effect on lifespan.”

3. The authors state that they did not study *arrd-12*, *22 *and *27* because of 'unknown technical problems'. It would be helpful for the field to be more specific here. Could they not be amplified?

> We appreciate your comments. We were not able to obtain transformed *E. coli* HT115 with infusion reaction products for *arrd-12* or *arrd-22* RNAi clones, or the PCR product for *arrd-27* genomic or complementary DNA. We specified the ‘unknown technical problems’ by mentioning these details in the main text as follows,

In Methods, RNAi clones: “RNAi clone for *arrd-12*, *arrd-22*, or *arrd-27* was not generated because we were not able to obtain transformed *E. coli* (HT115) colonies using *arrd-12* or *arrd-22* infusion reaction products, or the PCR product for *arrd-27* genomic or complementary DNA.”

4. In the results paragraph the authors state that 'knockdown of each of individual α-arrestin genes tended to slightly reduce lifespan in wild-type or in *daf-2(-) *mutants'

This is a little misleading. Change to 'knockdown of specific α-arrestin genes caused a minor reduction of lifespan in wild-type or in *daf-2(-)* mutants'

> We appreciate your comments. We changed the sentence as follows,

In Results: “We found that knockdown of specific α-arrestin genes caused a minor reduction of lifespan in wild-type or in *daf-2(-)* mutants (Figure 1B, Figure S1B-U, Table S2).”

5. Change from 'In addition, as we did not test whether RNAi targeting each of α-arrestin genes was effective by using quantitative RT-PCR' to 'In addition, as we did not test whether RNAi targeting of each α-arrestin gene was effective by using quantitative RT-PCR'

> We appreciate your comments. We changed the sentence as suggested.

In Discussion: “In addition, as we did not test whether RNAi targeting each α-arrestin gene was effective by using quantitative RT-PCR”

# Peer review: Referee response for version 1

**DOI:** 10.5256/f1000research.13357.r25722

**Published:** 2017-09-06

**Authors:** Roger Pocock

**Affiliations:** 1 Development and Stem Cells Program, Monash Biomedicine Discovery Institute, Department of Anatomy and Developmental Biology, Monash University, Melbourne, Vic, Australia

In this manuscript Park *et al*. performed RNAi knockdown of 24 of the 29 alpha-arrestin genes in C. elegans to ask whether they are important for lifespan regulation in wild type and daf-2 mutant animals. Their data shows that, in their experimental conditions, most alpha-arrestin genes are individually not important for regulating lifespan.

 Concerns:

 One major issue with the study is whether RNAi knockdown of the alpha-arrestin genes is working. As some alpha-arrestins are expressed in the nervous system, which is refractory to RNAi, this is a concern. To determine whether the RNAi knockdown is working then Q-RTPCR should be performed. However, this is not a small task for 24 genes, therefore, further experiments using sensitized genetic backgrounds for RNAi or elevated temperatures may tease out phenotypes for some alpha-arrestin genes. This could particularly be performed for the alpha-arrestin genes that when knocked down engender a slight effect on lifespan.

 In parallel to this, the authors could align the alpha-arrestin gene sequences to ask if there is a common sequence that could be used by RNAi targeting to knockdown multiple alpha-arrestin genes simultaneously.

 The authors knockdown of 24 of the 29 alpha-arrestin genes. I wonder why they didn’t generate RNAi clones for the remaining 5, especially since they did generate 3 other constructs for their analysis. Potentially, the important alpha-arrestin genes is amongst those not examined.

 The authors did not report any other behavioural or morphological phenotypes observed by alpha-arrestin gene knockdown. Any information on observed phenotypes would be of interest to the research community.

I have read this submission. I believe that I have an appropriate level of expertise to confirm that it is of an acceptable scientific standard, however I have significant reservations, as outlined above.

# Response

**Published:** 2017-10-16

**Authors:** Seung-Jae V Lee

**Affiliations:** Department of Life Sciences, Pohang University of Science and Technology, Korea, South

Dear Dr. Roger Pocock,

 We sincerely appreciate your valuable comments on our manuscript. We agree with all your points and revised our manuscript to describe the limitations of this study. We would like to mention that the main motivation for publishing this manuscript is to provide our negative results to researchers who are interested in characterizing α-arrestins in *C. elegans*. Our point-by-point responses are described below. We hope you find our revised manuscript is suitable for publication in *F1000Research*.

 In this manuscript Park et al. performed RNAi knockdown of 24 of the 29 alpha-arrestin genes in *C. elegans* to ask whether they are important for lifespan regulation in wild type and *daf-2* mutant animals. Their data shows that, in their experimental conditions, most alpha-arrestin genes are individually not important for regulating lifespan.

 Concerns:

 One major issue with the study is whether RNAi knockdown of the alpha-arrestin genes is working. As some alpha-arrestins are expressed in the nervous system, which is refractory to RNAi, this is a concern. To determine whether the RNAi knockdown is working then Q-RTPCR should be performed. However, this is not a small task for 24 genes, therefore, further experiments using sensitized genetic backgrounds for RNAi or elevated temperatures may tease out phenotypes for some alpha-arrestin genes. This could particularly be performed for the alpha-arrestin genes that when knocked down engender a slight effect on lifespan.

 > We agree with the reviewer’s comment that the RNAi knockdown efficiency is a major concern in interpreting our screen results. It would have been great if we had performed qRT-PCR analysis. However, as the reviewers pointed out, this is not a small task. We currently do not have any plan for following up this project and want to inform other researchers of our screen data. Therefore, we further emphasized the pitfall of our results in the Discussion as follows,

 In Discussion: “The lifespan-regulatory functions of α-arrestins remain largely unexplored at the organism level. Here we showed that RNAi knockdown of individual *C. elegans* α-arrestins had small or no effects on lifespan in wild-type or *daf-2* mutants. Our study has limitations that need to be considered for interpretation. First, RNAi targeting some α-arrestins might be insufficient for causing strong lifespan phenotypes. This may be because many of *C. elegans* α-arrestins are predicted to be expressed in neurons ^7, 9, 24-26^, which are refractory to RNAi ^27-29^. Lifespan assays using RNAi-hypersensitive mutants, including *rrf-3(-)* and *eri-1(-)* animals ^29-31^, treated with α-arrestin RNAi, or using α-arrestin null mutants will help address this issue. In addition, as we did not test whether RNAi targeting each of α-arrestin genes was effective by using quantitative RT-PCR, our negative data should be interpreted with caution.”

 In parallel to this, the authors could align the alpha-arrestin gene sequences to ask if there is a common sequence that could be used by RNAi targeting to knockdown multiple alpha-arrestin genes simultaneously.

 > We appreciate the reviewer’s comment. The α-arrestin family members share arrestin-domains (O. Hobert, *Wormbook*, 2013). Thus, it will be intriguing if we could examine whether targeting arrestin domain for RNAi to knock down multiple *arrd* genes simultaneously and if multiple *arrd* gene knock down has effects on lifespan. We modified our manuscript to address this issue in Discussion as follows,

 In Discussion: “Second, *C. elegans* α-arrestins may have functional redundancy considering the large number of the α-arrestin family members in *C. elegans* and their sequence similarity ^1, 7^, which may obscure examining the functional importance of each α-arrestin. In addition, some α-arrestins may mostly function by modulating their interacting proteins ^6^. In this case, genetic inhibition of α-arrestins may rather subtly affect the functions of their interacting partners that directly regulate physiology, such as aging and longevity, causing weak or no phenotypes. Thus, it will be interesting to test the effects of simultaneous inhibition of α-arrestins possibly through targeting the arrestin domain, and to identify and to functionally characterize proteins that bind *C. elegans* α-arrestins.”

 The authors knockdown of 24 of the 29 alpha-arrestin genes. I wonder why they didn’t generate RNAi clones for the remaining 5, especially since they did generate 3 other constructs for their analysis. Potentially, the important alpha-arrestin genes is amongst those not examined.

 > We appreciate the reviewers’ comment. We aimed at obtaining all the α arrestin RNAi clones that are not commercially available, except two predicted pseudogenes, *arrd-20* and *arrd-21* (Wormbase, version WS259). We successfully constructed three RNAi clones targeting *arrd-11*, *arrd-17* and *arrd-26*, but could not obtain the other three RNAi clones, targeting *arrd-12*, *arrd-22* and *arrd-27*. We addressed this issue in Methods for RNAi clones and in Discussion as follows,

 In Methods, RNAi clones: “RNAi clones for *arrd-12*, *arrd-22*, and *arrd-27* were not generated because of unknown technical problems and were not included in the lifespan screen. *arrd-20* and *arrd-21* are predicted to be pseudogenes (Wormbase, version WS259) and were excluded from our screen.”

 In Discussion: “Third, it is possible that three α-arrestins, *arrd-12*, *arrd-22* and *arrd-27*, which were not tested in our screen, may play crucial roles in lifespan regulation. Thus, it will be important to examine if genetic inhibition of each of these three α-arrestin genes affects lifespan in future studies.”

 The authors did not report any other behavioural or morphological phenotypes observed by alpha-arrestin gene knockdown. Any information on observed phenotypes would be of interest to the research community.

 > We thank the reviewer for the valuable comment. One noticeable phenotype we observed was that the eggs laid by worms fed with *ttm-2* RNAi bacteria hatched more frequently than control eggs on FUdR-treated plates, although we did not quantify the results. We described this issue in Methods as follows,

 In Methods, RNAi screen using lifespan assays: “Eggs laid by *ttm-2* RNAi-treated worms hatched more frequently than control eggs on FUdR-containing plates in two independent lifespan experiments.”

# Peer review: Referee response for version 1

**DOI:** 10.5256/f1000research.13357.r25290

**Published:** 2017-09-04

**Authors:** Alessandro Bitto, Jason Pitt

**Affiliations:** 1 Department of Pathology, University of Washington, Seattle, Seattle, WA, USA; 2 Department of Pathology, University of Washington, Seattle, WA, USA

In the manuscript *RNAi targeting Caenorhabditis elegans alpha-arrestins marginally affects lifespan *Park and co-workers provide largely negative results from lifespan experiments following RNAi knockdown of most of the worm alpha-arrestins.  While we welcome the effort to publish negative results, there is one major concern with the experimental approach and several minor concerns with the interpretation of the results that should be addressed before the manuscript is fully accepted for indexing in *F1000Research*.

 Major concern:

 The authors data is almost entirely negative: there were only 4 clones which had a statistically significant effect.  The authors attempt to address this concern by providing RNAi data for daf-16, which is known to strongly suppress the lifespan extension of daf-2(e1370) as well as decreasing N2 lifespan.  However, the authors must demonstrate that alpha-arrestin RNAi knockdown is functioning.  As suggested by the authors, there is some evidence that alpha-arrestins are expressed in neurons which are notoriously refractory to RNAi.  The authors must show that they can use RNAi to knockdown alpha-arrestins by QRTPCR analysis.  The authors could also address this concern by performing the RNAi in one of the mutant strains in which RNAi works in neurons and report if the lifespan still does not change, as they suggest in the discussion. We believe that providing these data is necessary to correctly interpret the results from the lifespan analyses. 

 Minor concerns:

   

 1.  In the text the authors speak broadly of individual arrestins having no role in longevity however the authors only tested 24 of the 29 arrestins in the worm.  If the authors had limited themselves to only those clones available in commercial RNAi libraries this would be understandable, however the authors did construct 3 new RNAi feeding constructs.  This brings up the question to why the authors did not prepare the remaining 5 constructs.  If the authors have reason to exclude those genes from their analysis (ie. they are pseudogenes, not expressed, etc) they should discuss these reasons in the text.  Besides, there is the possibility that one of the missing constructs may indeed have major effects on lifespan in *C. elegans*. 

 2.  The title of the manuscript implies there are more than one result than was observed.  The simpler conclusion from the results provided is that individual alpha-arrestin knockdown has no effect of lifespan.  This is the fundamental observation of the paper considering that most of them (20 out of 24) had no effect and that 4 of them had such an extremely small effect that it is arguable that the sample size is insufficient to support any conclusion ^1^.  We understand the authors' motivation to make the manuscript sound more appealing, however, their interpretation is disingenuous, given their results.

 3.  Because alpha-arrestins share significant sequence homology the authors should make some mention as to the likelihood that RNAi of any one arrestin would have off target effects on other arrestins.  The authors could easily address this question experimentally with QRTPCR analysis. This is particularly relevant as one of the main conclusions of the manuscript is that future alpha-arrestin studies should knockdown multiple alpha-arrestins simultaneously.

 4.  Given that the authors observed the RNAi treated animals for weeks or months (in daf-2) it is surprising that no other behavioral or physical phenotypes from the alpha-arrestin RNAi were observed during the course of the analysis.  If the authors truly did not observe any other phenotypes they should state it briefly in the text.

We have read this submission. We believe that we have an appropriate level of expertise to confirm that it is of an acceptable scientific standard, however we have significant reservations, as outlined above.

## References

[1] PetrascheckMMillerDL: Computational Analysis of Lifespan Experiment Reproducibility. *Front Genet*.2017;8: 10.3389/fgene.2017.00092 92 10.3389/fgene.2017.00092 28713422PMC5492194

# Response

**Published:** 2017-10-16

**Authors:** Seung-Jae V Lee

**Affiliations:** Department of Life Sciences, Pohang University of Science and Technology, Korea, South

Dear Dr. Alessandro Bitto and Dr. Jason Pitt,

 We greatly appreciate the reviewers’ valuable comments on our manuscript. We agree with all the reviewers’ comments and modified our manuscript to describe the limitations of our studies raised by the reviewers. We would like to mention that the main purpose of publishing our current manuscript is to provide our data to other researchers who are interested in the characterization of *C. elegans* *arrd* genes, instead of keeping our current data unpublished. Our point-by-point responses are described below. We hope you find our revised manuscript is suitable for publication in *F1000Research*.

 Reviewers’ comments:

 In the manuscript RNAi targeting *Caenorhabditis elegans* alpha-arrestins marginally affects lifespan Park and co-workers provide largely negative results from lifespan experiments following RNAi knockdown of most of the worm alpha-arrestins. While we welcome the effort to publish negative results, there is one major concern with the experimental approach and several minor concerns with the interpretation of the results that should be addressed before the manuscript is fully accepted for indexing in *F1000Research*.

 Major concern:

 The authors data is almost entirely negative: there were only 4 clones which had a statistically significant effect. The authors attempt to address this concern by providing RNAi data for *daf-16*, which is known to strongly suppress the lifespan extension of *daf-2(e1370)* as well as decreasing N2 lifespan. However, the authors must demonstrate that alpha-arrestin RNAi knockdown is functioning. As suggested by the authors, there is some evidence that alpha-arrestins are expressed in neurons which are notoriously refractory to RNAi. The authors must show that they can use RNAi to knockdown alpha-arrestins by QRTPCR analysis. The authors could also address this concern by performing the RNAi in one of the mutant strains in which RNAi works in neurons and report if the lifespan still does not change, as they suggest in the discussion. We believe that providing these data is necessary to correctly interpret the results from the lifespan analyses.

 > We completely agree with the reviewers for the comment. It would have been great if we had performed qRTPCR for validating the RNAi efficiency, while we were doing lifespan assays. As our experiments were done as an RNAi screen, our initial plan was validating RNAi efficiency and using RNAi-sensitive mutants if we had identified some positive RNAi clones. However, as the reviewers pointed out, our screen results were negative. We currently do not have any plan for following up this project and just want to inform other researchers of our screen data. Therefore, instead of putting efforts on this project retrospectively, we emphasized the limitations of our data raised by the reviewers to inform the readers of pitfalls by revising the manuscript as follows,

 In Discussion: “The lifespan-regulatory functions of α-arrestins remain largely unexplored at the organism level. Here we showed that RNAi knockdown of individual *C. elegans* α-arrestins had small or no effects on lifespan in wild-type or *daf-2* mutants. Our study has limitations that need to be considered for interpretation. First, RNAi targeting some α-arrestins might be insufficient for causing strong lifespan phenotypes. This may be because many of *C. elegans* α-arrestins are predicted to be expressed in neurons ^7, 9, 24-26^, which are refractory to RNAi ^27-29^. Lifespan assays using RNAi-hypersensitive mutants, including *rrf-3(-)* and *eri-1(-)* animals ^29-31^, treated with α-arrestin RNAi, or using α-arrestin null mutants will help address this issue. In addition, as we did not test whether RNAi targeting each of α-arrestin genes was effective by using quantitative RT-PCR, our negative data should be interpreted with caution.”

 Minor concerns:

 1. In the text the authors speak broadly of individual arrestins having no role in longevity however the authors only tested 24 of the 29 arrestins in the worm. If the authors had limited themselves to only those clones available in commercial RNAi libraries this would be understandable, however the authors did construct 3 new RNAi feeding constructs. This brings up the question to why the authors did not prepare the remaining 5 constructs. If the authors have reason to exclude those genes from their analysis (ie. they are pseudogenes, not expressed, etc) they should discuss these reasons in the text. Besides, there is the possibility that one of the missing constructs may indeed have major effects on lifespan in *C. elegans*.

 > We appreciate the reviewers’ comment. We initially aimed at obtaining all the α arrestin RNAi clones that are not commercially available except two predicted pseudogenes, *arrd-20* and *arrd-21* (Wormbase, version WS259). We successfully constructed three RNAi clones targeting *arrd-11*, *arrd-17* and *arrd-26*, but could not obtain the other three RNAi clones for *arrd-12*, *arrd-22* and *arrd-27* because of unknown technical problems. We agree with the reviewers’ point that these three genes that were not tested may have major effects on lifespan. We addressed this issue in Methods for RNAi clones as well as in Discussion as follows,

 In Methods, RNAi clones: “RNAi clones for *arrd-12*, *arrd-22*, and *arrd-27* were not generated because of unknown technical problems and were not included in the lifespan screen. *arrd-20* and *arrd-21* are predicted to be pseudogenes (Wormbase, version WS259) and were excluded from our screen.”

 In Discussion: “Third, it is possible that three α-arrestins, *arrd-12*, *arrd-22* and *arrd-27*, which were not tested in our screen, may play crucial roles in lifespan regulation. Thus, it will be important to examine if genetic inhibition of each of these three α-arrestin genes affects lifespan in future studies.”

 2. The title of the manuscript implies there are more than one result than was observed. The simpler conclusion from the results provided is that individual alpha-arrestin knockdown has no effect of lifespan. This is the fundamental observation of the paper considering that most of them (20 out of 24) had no effect and that 4 of them had such an extremely small effect that it is arguable that the sample size is insufficient to support any conclusion ^1^. We understand the authors' motivation to make the manuscript sound more appealing, however, their interpretation is disingenuous, given their results.

 > We thank the reviewers’ comment on the title and changed the title as follows,

 Title: “RNAi targeting *Caenorhabditis elegans *α-arrestins has small or no effects on lifespan”

 3. Because alpha-arrestins share significant sequence homology the authors should make some mention as to the likelihood that RNAi of any one arrestin would have off target effects on other arrestins. The authors could easily address this question experimentally with QRTPCR analysis. This is particularly relevant as one of the main conclusions of the manuscript is that future alpha-arrestin studies should knockdown multiple alpha-arrestins simultaneously.

 > We agree with the reviewers that the RNAi targeting each of α-arrestin genes may have caused off-target effects on other α-arrestin genes because of sequence similarities. We performed off-target prediction using Clone Mapper (http://bioinformatics.lif.univ-mrs.fr/RNAiMap), and at least with the prediction, the RNAi clones used in our screen do not appear to have major off-target effects on any other genes including α-arrestin genes, except *arrd-19* RNAi which may also target *arrd-21* which is a predicted pseudogene. Although qRT-PCR will be a straightforward way as the reviewers suggested, again currently we do not have any plan on following up this project and we therefore instead acknowledged the limitation. We added the prediction results in the Supplementary Table S1 and described this issue in the Methods, RNAi clones part as follows,

 In Methods, RNAi clones: “All RNAi clones used in our screen were examined for their potential off-target effects by using Clone Mapper (http://bioinformatics.lif.univ-mrs.fr/RNAiMap) ^22^, and no significant off-target was predicted except *arrd-19* RNAi, which may additionally target a predicted pseudogene *arrd-21* (Table S1). Experimental validation by qRT-PCR will be necessary to completely exclude the possible off-target effects by RNAi clones.”

 In Supplementary material: “Table S1. Possible RNAi target prediction. RNAi clones used in the screen were examined for their possible off-targets by Clone Mapper (http://bioinformatics.lif.univ-mrs.fr/RNAiMap) ^22^. For RNAi clone names, ‘mv’ indicates a RNAi clone from Vidal library and ‘sjj’ indicates a RNAi clone from Ahringer library. RNAi clones generated by us for this study are labeled as ‘Lee lab’. Scores for potential target transcripts were calculated as described previously ^22^ and were rounded off to the second digit after the decimal point.”

 In Reference: 22. Thakur, N., et al., Clone Mapper: An Online Suite of Tools for RNAi Experiments in *Caenorhabditis elegans*. G3 (Bethesda), 2014. 4(11): p. 2137-2145.23.

 4. Given that the authors observed the RNAi treated animals for weeks or months (in *daf-2*) it is surprising that no other behavioral or physical phenotypes from the alpha-arrestin RNAi were observed during the course of the analysis. If the authors truly did not observe any other phenotypes they should state it briefly in the text.

 > We appreciate the reviewers’ comment. As our main interests were lifespan changes from a large scale screen, we did not pay attention to other phenotypes. Nevertheless, one noticeable phenotype we observed was that the eggs laid by worms fed with *ttm-2* RNAi bacteria hatched more frequently than control eggs on FUdR-treated plates, although we did not quantitate the results. We described this issue in Methods as follows,

 In Methods, RNAi screen using lifespan assays: “Eggs laid by *ttm-2* RNAi-treated worms hatched more frequently than control eggs on FUdR-containing plates in two independent lifespan experiments.”
